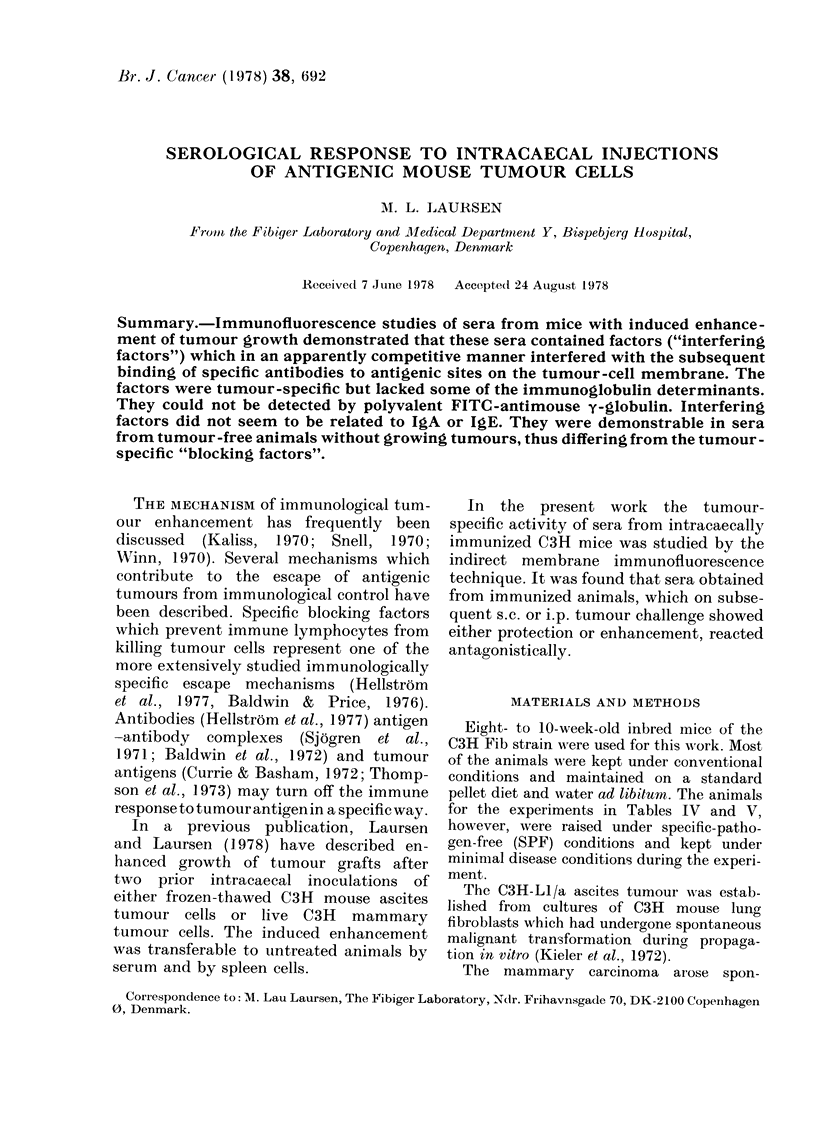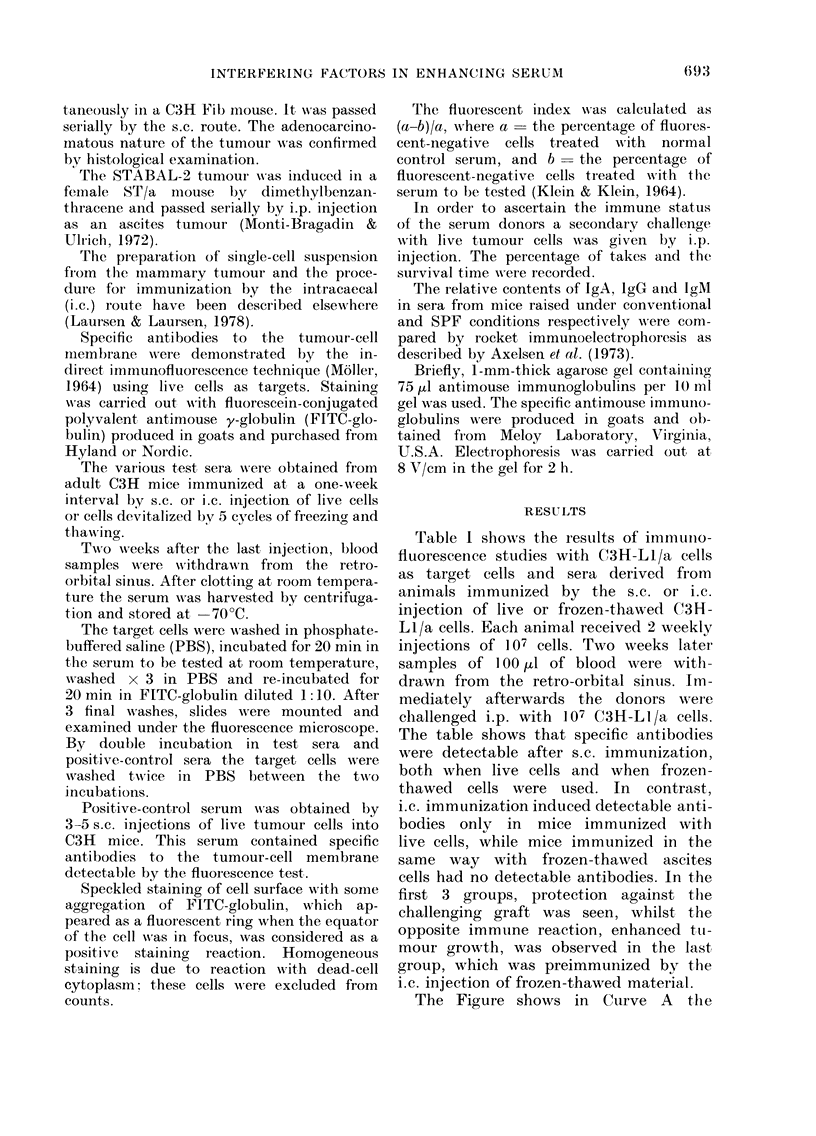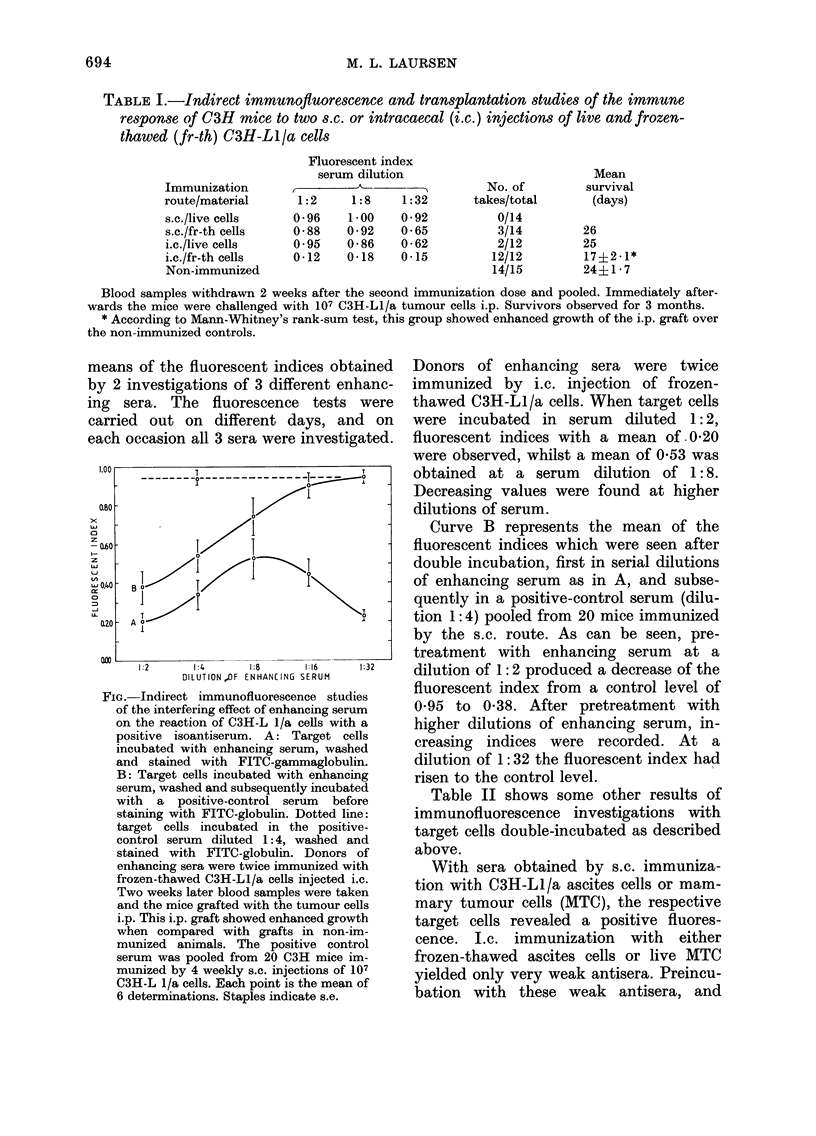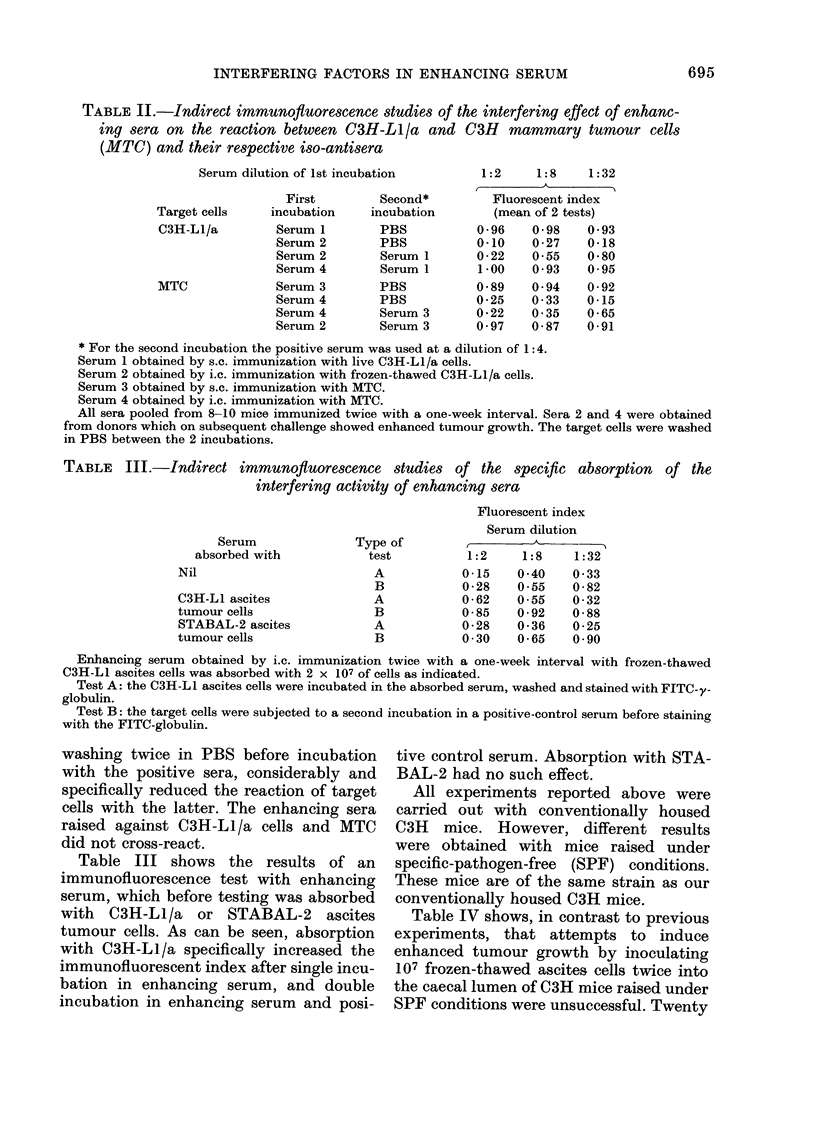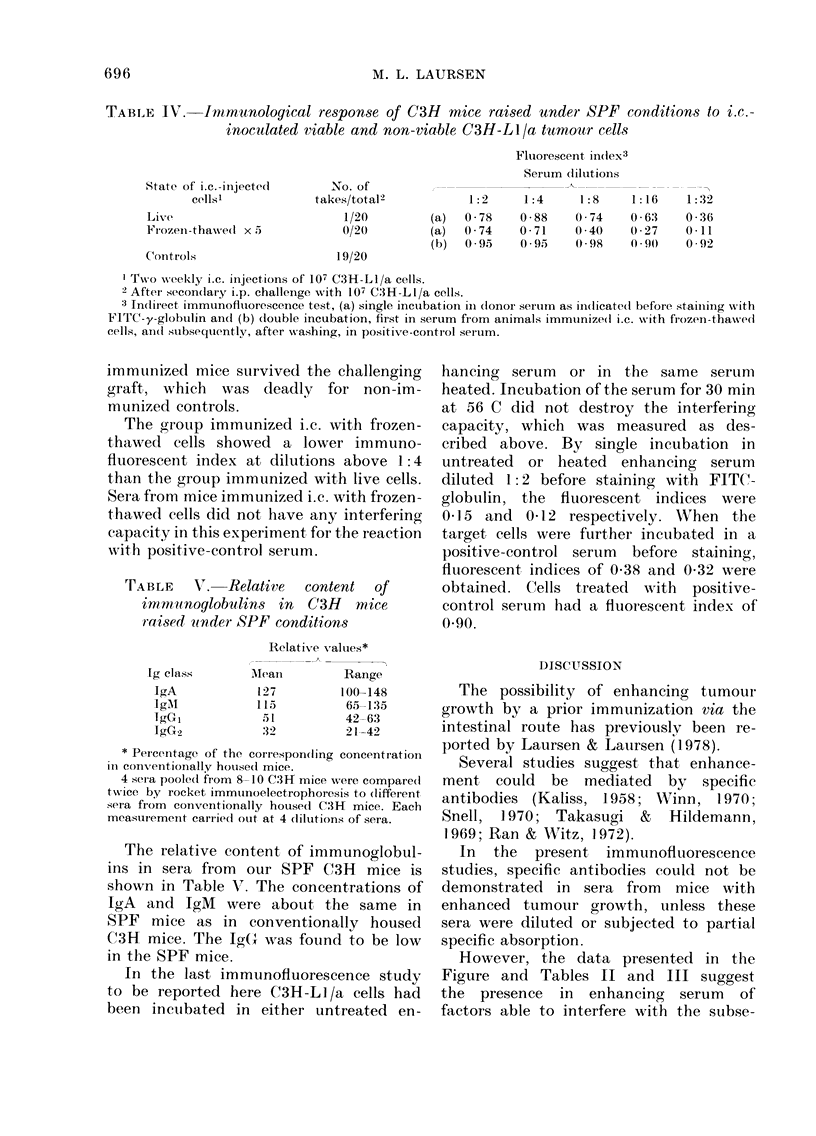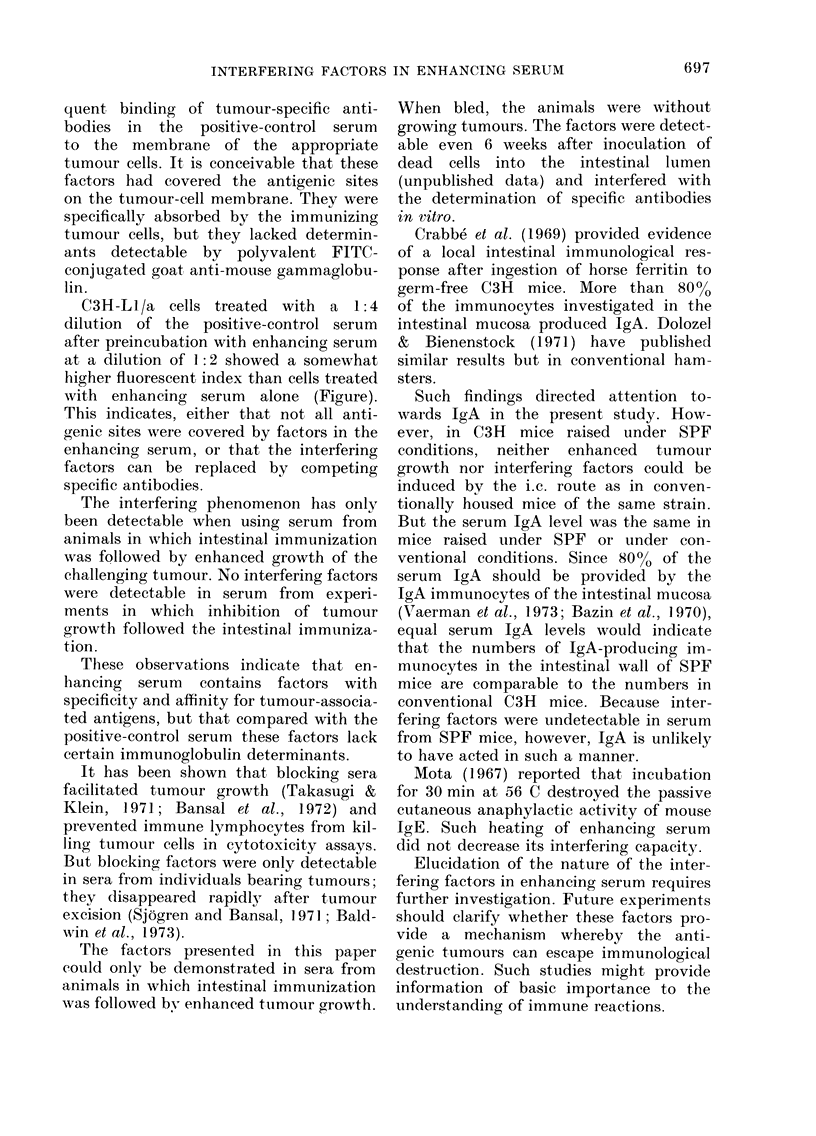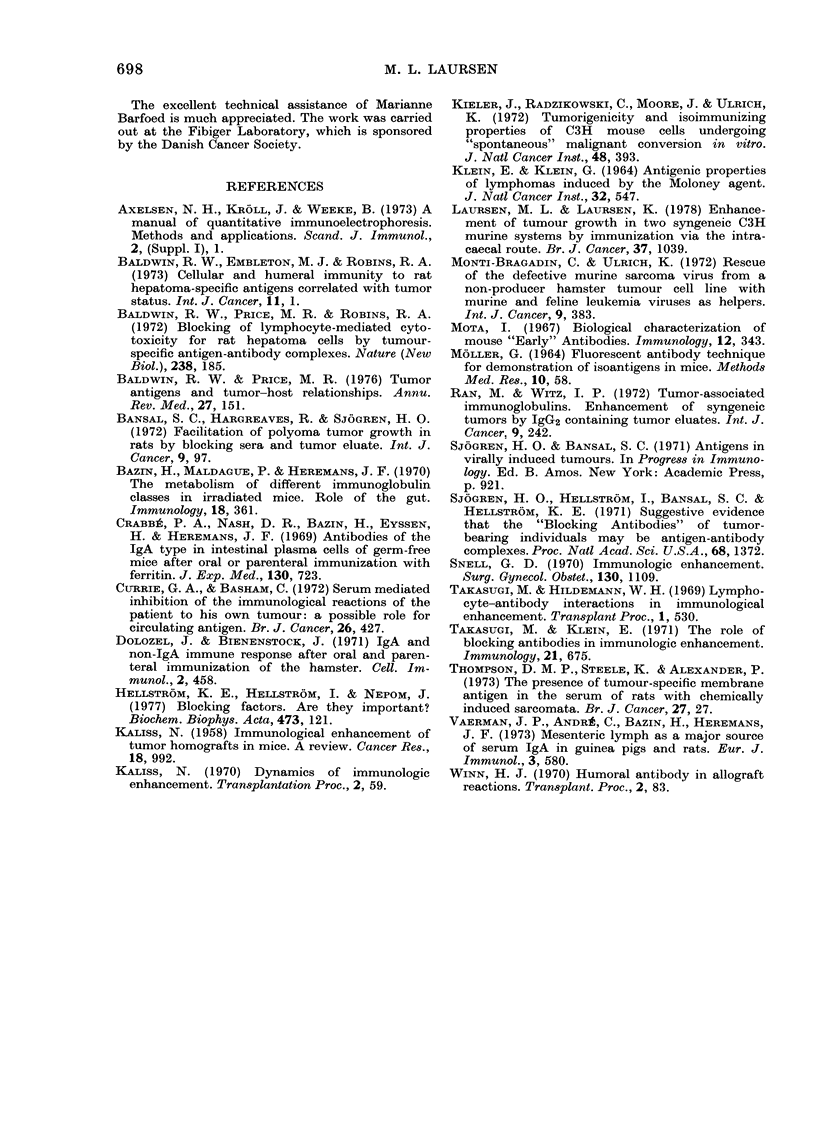# Serological response to intracaecal injections of antigenic mouse tumour cells.

**DOI:** 10.1038/bjc.1978.274

**Published:** 1978-12

**Authors:** M. L. Laursen

## Abstract

Immunofluorescence studies of sera from mice with induced enhancement of tumour growth demonstrated that these sera contained factors ("interfering factors") which in an apparently competitive manner interfered with the subsequent binding of specific antibodies to antigenic sites on the tumour-cell membrane. The factors were tumour-specific but lacked some of the immunoglobulin determinants. They could not be detected by polyvalent FITC-antimouse gamma-globulin. Interfering factors did not seem to be related to IgA or IgE. They were demonstrable in sera from tumour-free animals without growing tumours, thus differing from the tumour-specific "blocking factors".


*Br. J. Cancer* (1978) **38**, 692

### SEROLOGICAL RESPONSE TO INTRACAECAL INJECTIONS OF ANTIGENIC MOUSE TUMOUR CELLS

M. L. LAURSEN

*From the Fibiger Laboratory and Medical Department Y, Bispebjerg Hospital, Copenhagen, Denmark*

Received 7 June 1978 Accepted 24 August 1978

**Summary.—Immunofluorescence studies of sera from mice with induced enhancement of tumour growth demonstrated that these sera contained factors ("interfering factors") which in an apparently competitive manner interfered with the subsequent binding of specific antibodies to antigenic sites on the tumour-cell membrane. The factors were tumour-specific but lacked some of the immunoglobulin determinants. They could not be detected by polyvalent FITC-antimouse γ-globulin. Interfering factors did not seem to be related to IgA or IgE. They were demonstrable in sera from tumour-free animals without growing tumours, thus differing from the tumour-specific "blocking factors".**

THE MECHANISM of immunological tumour enhancement has frequently been discussed (Kaliss, 1970; Snell, 1970; Winn, 1970). Several mechanisms which contribute to the escape of antigenic tumours from immunological control have been described. Specific blocking factors which prevent immune lymphocytes from killing tumour cells represent one of the more extensively studied immunologically specific escape mechanisms (Hellström *et al.*, 1977, Baldwin & Price, 1976). Antibodies (Hellström *et al.*, 1977) antigen –antibody complexes (Sjögren *et al.*, 1971; Baldwin *et al.*, 1972) and tumour antigens (Currie & Basham, 1972; Thompson *et al.*, 1973) may turn off the immune response to tumour antigen in a specific way.

In a previous publication, Laursen and Laursen (1978) have described enhanced growth of tumour grafts after two prior intracaecal inoculations of either frozen-thawed C3H mouse ascites tumour cells or live C3H mammary tumour cells. The induced enhancement was transferable to untreated animals by serum and by spleen cells.

In the present work the tumour-specific activity of sera from intracaecally immunized C3H mice was studied by the indirect membrane immunofluorescence technique. It was found that sera obtained from immunized animals, which on subsequent s.c. or i.p. tumour challenge showed either protection or enhancement, reacted antagonistically.

### MATERIALS AND METHODS

Eight- to 10-week-old inbred mice of the C3H Fib strain were used for this work. Most of the animals were kept under conventional conditions and maintained on a standard pellet diet and water *ad libitum*. The animals for the experiments in Tables IV and V, however, were raised under specific-pathogen-free (SPF) conditions and kept under minimal disease conditions during the experiment.

The C3H-L1/a ascites tumour was established from cultures of C3H mouse lung fibroblasts which had undergone spontaneous malignant transformation during propagation *in vitro* (Kieler *et al.*, 1972).

The mammary carcinoma arose spon-

Correspondence to: M. Lau Laursen, The Fibiger Laboratory, Ndr. Frihavnsgade 70, DK-2100 Copenhagen Ø, Denmark.

taneously in a C3H Fib mouse. It was passed serially by the s.c. route. The adenocarcinomatous nature of the tumour was confirmed by histological examination.

The STABAL-2 tumour was induced in a female ST/a mouse by dimethylbenzanthracene and passed serially by i.p. injection as an ascites tumour (Monti-Bragadin & Ulrich, 1972).

The preparation of single-cell suspension from the mammary tumour and the procedure for immunization by the intracaecal (i.c.) route have been described elsewhere (Laursen & Laursen, 1978).

Specific antibodies to the tumour-cell membrane were demonstrated by the indirect immunofluorescence technique (Möller, 1964) using live cells as targets. Staining was carried out with fluorescein-conjugated polyvalent antimouse $\gamma$-globulin (FITC-globulin) produced in goats and purchased from Hyland or Nordic.

The various test sera were obtained from adult C3H mice immunized at a one-week interval by s.c. or i.c. injection of live cells or cells devitalized by 5 cycles of freezing and thawing.

Two weeks after the last injection, blood samples were withdrawn from the retro-orbital sinus. After clotting at room temperature the serum was harvested by centrifugation and stored at $-70$°C.

The target cells were washed in phosphate-buffered saline (PBS), incubated for 20 min in the serum to be tested at room temperature, washed $\times$ 3 in PBS and re-incubated for 20 min in FITC-globulin diluted 1:10. After 3 final washes, slides were mounted and examined under the fluorescence microscope. By double incubation in test sera and positive-control sera the target cells were washed twice in PBS between the two incubations.

Positive-control serum was obtained by 3–5 s.c. injections of live tumour cells into C3H mice. This serum contained specific antibodies to the tumour-cell membrane detectable by the fluorescence test.

Speckled staining of cell surface with some aggregation of FITC-globulin, which appeared as a fluorescent ring when the equator of the cell was in focus, was considered as a positive staining reaction. Homogeneous staining is due to reaction with dead-cell cytoplasm; these cells were excluded from counts.

The fluorescent index was calculated as $(a-b)/a$, where $a$ = the percentage of fluorescent-negative cells treated with normal control serum, and $b$ = the percentage of fluorescent-negative cells treated with the serum to be tested (Klein & Klein, 1964).

In order to ascertain the immune status of the serum donors a secondary challenge with live tumour cells was given by i.p. injection. The percentage of takes and the survival time were recorded.

The relative contents of IgA, IgG and IgM in sera from mice raised under conventional and SPF conditions respectively were compared by rocket immunoelectrophoresis as described by Axelsen *et al.* (1973).

Briefly, 1-mm-thick agarose gel containing 75 $\mu$l antimouse immunoglobulins per 10 ml gel was used. The specific antimouse immunoglobulins were produced in goats and obtained from Meloy Laboratory, Virginia, U.S.A. Electrophoresis was carried out at 8 V/cm in the gel for 2 h.

### RESULTS

Table I shows the results of immunofluorescence studies with C3H-L1/a cells as target cells and sera derived from animals immunized by the s.c. or i.c. injection of live or frozen-thawed C3H-L1/a cells. Each animal received 2 weekly injections of $10^7$ cells. Two weeks later samples of 100 $\mu$l of blood were withdrawn from the retro-orbital sinus. Immediately afterwards the donors were challenged i.p. with $10^7$ C3H-L1/a cells. The table shows that specific antibodies were detectable after s.c. immunization, both when live cells and when frozen-thawed cells were used. In contrast, i.c. immunization induced detectable antibodies only in mice immunized with live cells, while mice immunized in the same way with frozen-thawed ascites cells had no detectable antibodies. In the first 3 groups, protection against the challenging graft was seen, whilst the opposite immune reaction, enhanced tumour growth, was observed in the last group, which was preimmunized by the i.c. injection of frozen-thawed material.

The Figure shows in Curve A the

TABLE I.—*Indirect immunofluorescence and transplantation studies of the immune response of C3H mice to two s.c. or intracaecal (i.c.) injections of live and frozen-thawed (fr-th) C3H-L1/a cells*

| Immunization route/material | Fluorescent index serum dilution 1:2 | 1:8 | 1:32 | No. of takes/total | Mean survival (days) |
|---|---|---|---|---|---|
| s.c./live cells | 0·96 | 1·00 | 0·92 | 0/14 | |
| s.c./fr-th cells | 0·88 | 0·92 | 0·65 | 3/14 | 26 |
| i.c./live cells | 0·95 | 0·86 | 0·62 | 2/12 | 25 |
| i.c./fr-th cells | 0·12 | 0·18 | 0·15 | 12/12 | 17±2·1* |
| Non-immunized | | | | 14/15 | 24±1·7 |

Blood samples withdrawn 2 weeks after the second immunization dose and pooled. Immediately afterwards the mice were challenged with $10^7$ C3H-L1/a tumour cells i.p. Survivors observed for 3 months.
* According to Mann-Whitney's rank-sum test, this group showed enhanced growth of the i.p. graft over the non-immunized controls.

means of the fluorescent indices obtained by 2 investigations of 3 different enhancing sera. The fluorescence tests were carried out on different days, and on each occasion all 3 sera were investigated.

FIG.—Indirect immunofluorescence studies of the interfering effect of enhancing serum on the reaction of C3H-L 1/a cells with a positive isoantiserum. A: Target cells incubated with enhancing serum, washed and stained with FITC-gammaglobulin. B: Target cells incubated with enhancing serum, washed and subsequently incubated with a positive-control serum before staining with FITC-globulin. Dotted line: target cells incubated in the positive-control serum diluted 1:4, washed and stained with FITC-globulin. Donors of enhancing sera were twice immunized with frozen-thawed C3H-L1/a cells injected i.c. Two weeks later blood samples were taken and the mice grafted with the tumour cells i.p. This i.p. graft showed enhanced growth when compared with grafts in non-immunized animals. The positive control serum was pooled from 20 C3H mice immunized by 4 weekly s.c. injections of $10^7$ C3H-L 1/a cells. Each point is the mean of 6 determinations. Staples indicate s.e.

Donors of enhancing sera were twice immunized by i.c. injection of frozen-thawed C3H-L1/a cells. When target cells were incubated in serum diluted 1:2, fluorescent indices with a mean of 0·20 were observed, whilst a mean of 0·53 was obtained at a serum dilution of 1:8. Decreasing values were found at higher dilutions of serum.

Curve B represents the mean of the fluorescent indices which were seen after double incubation, first in serial dilutions of enhancing serum as in A, and subsequently in a positive-control serum (dilution 1:4) pooled from 20 mice immunized by the s.c. route. As can be seen, pretreatment with enhancing serum at a dilution of 1:2 produced a decrease of the fluorescent index from a control level of 0·95 to 0·38. After pretreatment with higher dilutions of enhancing serum, increasing indices were recorded. At a dilution of 1:32 the fluorescent index had risen to the control level.

Table II shows some other results of immunofluorescence investigations with target cells double-incubated as described above.

With sera obtained by s.c. immunization with C3H-L1/a ascites cells or mammary tumour cells (MTC), the respective target cells revealed a positive fluorescence. I.c. immunization with either frozen-thawed ascites cells or live MTC yielded only very weak antisera. Preincubation with these weak antisera, and

TABLE II.—*Indirect immunofluorescence studies of the interfering effect of enhancing sera on the reaction between C3H-L1/a and C3H mammary tumour cells (MTC) and their respective iso-antisera*

| Serum dilution of 1st incubation | | | 1:2 | 1:8 | 1:32 |
|---|---|---|---|---|---|
| Target cells | First incubation | Second* incubation | Fluorescent index (mean of 2 tests) | | |
| C3H-L1/a | Serum 1 | PBS | 0·96 | 0·98 | 0·93 |
| | Serum 2 | PBS | 0·10 | 0·27 | 0·18 |
| | Serum 2 | Serum 1 | 0·22 | 0·55 | 0·80 |
| | Serum 4 | Serum 1 | 1·00 | 0·93 | 0·95 |
| MTC | Serum 3 | PBS | 0·89 | 0·94 | 0·92 |
| | Serum 4 | PBS | 0·25 | 0·33 | 0·15 |
| | Serum 4 | Serum 3 | 0·22 | 0·35 | 0·65 |
| | Serum 2 | Serum 3 | 0·97 | 0·87 | 0·91 |

* For the second incubation the positive serum was used at a dilution of 1:4.
Serum 1 obtained by s.c. immunization with live C3H-L1/a cells.
Serum 2 obtained by i.c. immunization with frozen-thawed C3H-L1/a cells.
Serum 3 obtained by s.c. immunization with MTC.
Serum 4 obtained by i.c. immunization with MTC.
All sera pooled from 8–10 mice immunized twice with a one-week interval. Sera 2 and 4 were obtained from donors which on subsequent challenge showed enhanced tumour growth. The target cells were washed in PBS between the 2 incubations.

TABLE III.—*Indirect immunofluorescence studies of the specific absorption of the interfering activity of enhancing sera*

| Serum absorbed with | Type of test | Fluorescent index — Serum dilution 1:2 | 1:8 | 1:32 |
|---|---|---|---|---|
| Nil | A | 0·15 | 0·40 | 0·33 |
| | B | 0·28 | 0·55 | 0·82 |
| C3H-L1 ascites | A | 0·62 | 0·55 | 0·32 |
| tumour cells | B | 0·85 | 0·92 | 0·88 |
| STABAL-2 ascites | A | 0·28 | 0·36 | 0·25 |
| tumour cells | B | 0·30 | 0·65 | 0·90 |

Enhancing serum obtained by i.c. immunization twice with a one-week interval with frozen-thawed C3H-L1 ascites cells was absorbed with 2 × $10^7$ of cells as indicated.
Test A: the C3H-L1 ascites cells were incubated in the absorbed serum, washed and stained with FITC-$\gamma$-globulin.
Test B: the target cells were subjected to a second incubation in a positive-control serum before staining with the FITC-globulin.

washing twice in PBS before incubation with the positive sera, considerably and specifically reduced the reaction of target cells with the latter. The enhancing sera raised against C3H-L1/a cells and MTC did not cross-react.

Table III shows the results of an immunofluorescence test with enhancing serum, which before testing was absorbed with C3H-L1/a or STABAL-2 ascites tumour cells. As can be seen, absorption with C3H-L1/a specifically increased the immunofluorescent index after single incubation in enhancing serum, and double incubation in enhancing serum and positive control serum. Absorption with STABAL-2 had no such effect.

All experiments reported above were carried out with conventionally housed C3H mice. However, different results were obtained with mice raised under specific-pathogen-free (SPF) conditions. These mice are of the same strain as our conventionally housed C3H mice.

Table IV shows, in contrast to previous experiments, that attempts to induce enhanced tumour growth by inoculating $10^7$ frozen-thawed ascites cells twice into the caecal lumen of C3H mice raised under SPF conditions were unsuccessful. Twenty

TABLE IV.—*Immunological response of C3H mice raised under SPF conditions to i.c.-inoculated viable and non-viable C3H-L1/a tumour cells*

| State of i.c.-injected cells[1] | No. of takes/total[2] | | Fluorescent index[3] Serum dilutions 1:2 | 1:4 | 1:8 | 1:16 | 1:32 |
|---|---|---|---|---|---|---|---|
| Live | 1/20 | (a) | 0·78 | 0·88 | 0·74 | 0·63 | 0·36 |
| Frozen-thawed ×5 | 0/20 | (a) | 0·74 | 0·71 | 0·40 | 0·27 | 0·11 |
| | | (b) | 0·95 | 0·95 | 0·98 | 0·90 | 0·92 |
| Controls | 19/20 | | | | | | |

[1] Two weekly i.c. injections of $10^7$ C3H-L1/a cells.
[2] After secondary i.p. challenge with $10^7$ C3H-L1/a cells.
[3] Indirect immunofluorescence test, (a) single incubation in donor serum as indicated before staining with FITC-γ-globulin and (b) double incubation, first in serum from animals immunized i.c. with frozen-thawed cells, and subsequently, after washing, in positive-control serum.

immunized mice survived the challenging graft, which was deadly for non-immunized controls.

The group immunized i.c. with frozen-thawed cells showed a lower immunofluorescent index at dilutions above 1:4 than the group immunized with live cells. Sera from mice immunized i.c. with frozen-thawed cells did not have any interfering capacity in this experiment for the reaction with positive-control serum.

TABLE V.—*Relative content of immunoglobulins in C3H mice raised under SPF conditions*

| Ig class | Relative values* Mean | Range |
|---|---|---|
| IgA | 127 | 100–148 |
| IgM | 115 | 65–135 |
| $IgG_1$ | 51 | 42–63 |
| $IgG_2$ | 32 | 21–42 |

* Percentage of the corresponding concentration in conventionally housed mice.
4 sera pooled from 8–10 C3H mice were compared twice by rocket immunoelectrophoresis to different sera from conventionally housed C3H mice. Each measurement carried out at 4 dilutions of sera.

The relative content of immunoglobulins in sera from our SPF C3H mice is shown in Table V. The concentrations of IgA and IgM were about the same in SPF mice as in conventionally housed C3H mice. The IgG was found to be low in the SPF mice.

In the last immunofluorescence study to be reported here C3H-L1/a cells had been incubated in either untreated enhancing serum or in the same serum heated. Incubation of the serum for 30 min at 56 C did not destroy the interfering capacity, which was measured as described above. By single incubation in untreated or heated enhancing serum diluted 1:2 before staining with FITC-globulin, the fluorescent indices were 0·15 and 0·12 respectively. When the target cells were further incubated in a positive-control serum before staining, fluorescent indices of 0·38 and 0·32 were obtained. Cells treated with positive-control serum had a fluorescent index of 0·90.

### DISCUSSION

The possibility of enhancing tumour growth by a prior immunization *via* the intestinal route has previously been reported by Laursen & Laursen (1978).

Several studies suggest that enhancement could be mediated by specific antibodies (Kaliss, 1958; Winn, 1970; Snell, 1970; Takasugi & Hildemann, 1969; Ran & Witz, 1972).

In the present immunofluorescence studies, specific antibodies could not be demonstrated in sera from mice with enhanced tumour growth, unless these sera were diluted or subjected to partial specific absorption.

However, the data presented in the Figure and Tables II and III suggest the presence in enhancing serum of factors able to interfere with the subsequent binding of tumour-specific antibodies in the positive-control serum to the membrane of the appropriate tumour cells. It is conceivable that these factors had covered the antigenic sites on the tumour-cell membrane. They were specifically absorbed by the immunizing tumour cells, but they lacked determinants detectable by polyvalent FITC-conjugated goat anti-mouse gammaglobulin.

C3H-L1/a cells treated with a 1:4 dilution of the positive-control serum after preincubation with enhancing serum at a dilution of 1:2 showed a somewhat higher fluorescent index than cells treated with enhancing serum alone (Figure). This indicates, either that not all antigenic sites were covered by factors in the enhancing serum, or that the interfering factors can be replaced by competing specific antibodies.

The interfering phenomenon has only been detectable when using serum from animals in which intestinal immunization was followed by enhanced growth of the challenging tumour. No interfering factors were detectable in serum from experiments in which inhibition of tumour growth followed the intestinal immunization.

These observations indicate that enhancing serum contains factors with specificity and affinity for tumour-associated antigens, but that compared with the positive-control serum these factors lack certain immunoglobulin determinants.

It has been shown that blocking sera facilitated tumour growth (Takasugi & Klein, 1971; Bansal *et al.*, 1972) and prevented immune lymphocytes from killing tumour cells in cytotoxicity assays. But blocking factors were only detectable in sera from individuals bearing tumours; they disappeared rapidly after tumour excision (Sjögren and Bansal, 1971; Baldwin *et al.*, 1973).

The factors presented in this paper could only be demonstrated in sera from animals in which intestinal immunization was followed by enhanced tumour growth. When bled, the animals were without growing tumours. The factors were detectable even 6 weeks after inoculation of dead cells into the intestinal lumen (unpublished data) and interfered with the determination of specific antibodies *in vitro*.

Crabbé *et al.* (1969) provided evidence of a local intestinal immunological response after ingestion of horse ferritin to germ-free C3H mice. More than 80% of the immunocytes investigated in the intestinal mucosa produced IgA. Dolozel & Bienenstock (1971) have published similar results but in conventional hamsters.

Such findings directed attention towards IgA in the present study. However, in C3H mice raised under SPF conditions, neither enhanced tumour growth nor interfering factors could be induced by the i.c. route as in conventionally housed mice of the same strain. But the serum IgA level was the same in mice raised under SPF or under conventional conditions. Since 80% of the serum IgA should be provided by the IgA immunocytes of the intestinal mucosa (Vaerman *et al.*, 1973; Bazin *et al.*, 1970), equal serum IgA levels would indicate that the numbers of IgA-producing immunocytes in the intestinal wall of SPF mice are comparable to the numbers in conventional C3H mice. Because interfering factors were undetectable in serum from SPF mice, however, IgA is unlikely to have acted in such a manner.

Mota (1967) reported that incubation for 30 min at 56 C destroyed the passive cutaneous anaphylactic activity of mouse IgE. Such heating of enhancing serum did not decrease its interfering capacity.

Elucidation of the nature of the interfering factors in enhancing serum requires further investigation. Future experiments should clarify whether these factors provide a mechanism whereby the antigenic tumours can escape immunological destruction. Such studies might provide information of basic importance to the understanding of immune reactions.

The excellent technical assistance of Marianne Barfoed is much appreciated. The work was carried out at the Fibiger Laboratory, which is sponsored by the Danish Cancer Society.

### REFERENCES

AXELSEN, N. H., KRÖLL, J. & WEEKE, B. (1973) A manual of quantitative immunoelectrophoresis. Methods and applications. *Scand. J. Immunol.*, **2**, (Suppl. I), 1.

BALDWIN, R. W., EMBLETON, M. J. & ROBINS, R. A. (1973) Cellular and humeral immunity to rat hepatoma-specific antigens correlated with tumor status. *Int. J. Cancer*, **11**, 1.

BALDWIN, R. W., PRICE, M. R. & ROBINS, R. A. (1972) Blocking of lymphocyte-mediated cytotoxicity for rat hepatoma cells by tumour-specific antigen-antibody complexes. *Nature* (*New Biol.*), **238**, 185.

BALDWIN, R. W. & PRICE, M. R. (1976) Tumor antigens and tumor–host relationships. *Annu. Rev. Med.*, **27**, 151.

BANSAL, S. C., HARGREAVES, R. & SJÖGREN, H. O. (1972) Facilitation of polyoma tumor growth in rats by blocking sera and tumor eluate. *Int. J. Cancer*, **9**, 97.

BAZIN, H., MALDAGUE, P. & HEREMANS, J. F. (1970) The metabolism of different immunoglobulin classes in irradiated mice. Role of the gut. *Immunology*, **18**, 361.

CRABBÉ, P. A., NASH, D. R., BAZIN, H., EYSSEN, H. & HEREMANS, J. F. (1969) Antibodies of the IgA type in intestinal plasma cells of germ-free mice after oral or parenteral immunization with ferritin. *J. Exp. Med.*, **130**, 723.

CURRIE, G. A., & BASHAM, C. (1972) Serum mediated inhibition of the immunological reactions of the patient to his own tumour: a possible role for circulating antigen. *Br. J. Cancer*, **26**, 427.

DOLOZEL, J. & BIENENSTOCK, J. (1971) IgA and non-IgA immune response after oral and parenteral immunization of the hamster. *Cell. Immunol.*, **2**, 458.

HELLSTRÖM, K. E., HELLSTRÖM, I. & NEPOM, J. (1977) Blocking factors. Are they important? *Biochem. Biophys. Acta*, **473**, 121.

KALISS, N. (1958) Immunological enhancement of tumor homografts in mice. A review. *Cancer Res.*, **18**, 992.

KALISS, N. (1970) Dynamics of immunologic enhancement. *Transplantation Proc.*, **2**, 59.

KIELER, J., RADZIKOWSKI, C., MOORE, J. & ULRICH, K. (1972) Tumorigenicity and isoimmunizing properties of C3H mouse cells undergoing "spontaneous" malignant conversion *in vitro*. *J. Natl Cancer Inst.*, **48**, 393.

KLEIN, E. & KLEIN, G. (1964) Antigenic properties of lymphomas induced by the Moloney agent. *J. Natl Cancer Inst.*, **32**, 547.

LAURSEN, M. L. & LAURSEN, K. (1978) Enhancement of tumour growth in two syngeneic C3H murine systems by immunization via the intracaecal route. *Br. J. Cancer*, **37**, 1039.

MONTI-BRAGADIN, C. & ULRICH, K. (1972) Rescue of the defective murine sarcoma virus from a non-producer hamster tumour cell line with murine and feline leukemia viruses as helpers. *Int. J. Cancer*, **9**, 383.

MOTA, I. (1967) Biological characterization of mouse "Early" Antibodies. *Immunology*, **12**, 343.

MÖLLER, G. (1964) Fluorescent antibody technique for demonstration of isoantigens in mice. *Methods Med. Res.*, **10**, 58.

RAN, M. & WITZ, I. P. (1972) Tumor-associated immunoglobulins. Enhancement of syngeneic tumors by $IgG_2$ containing tumor eluates. *Int. J. Cancer*, **9**, 242.

SJÖGREN, H. O. & BANSAL, S. C. (1971) Antigens in virally induced tumours. In *Progress in Immunology*. Ed. B. Amos. New York: Academic Press, p. 921.

SJÖGREN, H. O., HELLSTRÖM, I., BANSAL, S. C. & HELLSTRÖM, K. E. (1971) Suggestive evidence that the "Blocking Antibodies" of tumor-bearing individuals may be antigen-antibody complexes. *Proc. Natl Acad. Sci. U.S.A.*, **68**, 1372.

SNELL, G. D. (1970) Immunologic enhancement. *Surg. Gynecol. Obstet.*, **130**, 1109.

TAKASUGI, M. & HILDEMANN, W. H. (1969) Lymphocyte–antibody interactions in immunological enhancement. *Transplant Proc.*, **1**, 530.

TAKASUGI, M. & KLEIN, E. (1971) The role of blocking antibodies in immunologic enhancement. *Immunology*, **21**, 675.

THOMPSON, D. M. P., STEELE, K. & ALEXANDER, P. (1973) The presence of tumour-specific membrane antigen in the serum of rats with chemically induced sarcomata. *Br. J. Cancer*, **27**, 27.

VAERMAN, J. P., ANDRÉ, C., BAZIN, H., HEREMANS, J. F. (1973) Mesenteric lymph as a major source of serum IgA in guinea pigs and rats. *Eur. J. Immunol.*, **3**, 580.

WINN, H. J. (1970) Humoral antibody in allograft reactions. *Transplant. Proc.*, **2**, 83.